# Supplementary material for: In Situ Grown Vertically Oriented Graphene Coating on Copper by Plasma-Enhanced CVD to Form Superhydrophobic Surface and Effectively Protect Corrosion
Source: Nanomaterials (Basel). 2022 Sep 15;12(18):3202. doi: 10.3390/nano12183202 (PMC9504450; doi:10.3390/nano12183202)
Supplement: Supplementary file 1 [file nanomaterials-12-03202-s001.zip › nanomaterials-1886803-supplementary.pdf]

In Situ Grown Vertically Oriented Graphene Coating on Copper by Plasma-Enhanced CVD to Form Superhydrophobic Surface and Effectively Protect Corrosion

Xiaohang Zheng, Yaqian Yang, Yi Xian, Heng Chen, Wei Cai *

Harbin Institute of Technology, School of Materials Science and Engineering, Harbin 150001, China
* Correspondence: weicai@hit.edu.cn

Table S1. Corrosion parameters obtained from PDS measurements for the Cu, Gs-Cu and VFG-Cu.

Sample	j_{corr} (A/cm^2)	E_{corr} (V)	v_{corr} (mm/year)
Cu	6.09×10^{-6}	–0.289	7.57×10^{-2}
GS-Cu	7.09×10^{-7}	–0.209	8.21×10^{-3}
VFG-Cu	7.90×10^{-8}	–0.125	9.14×10^{-4}

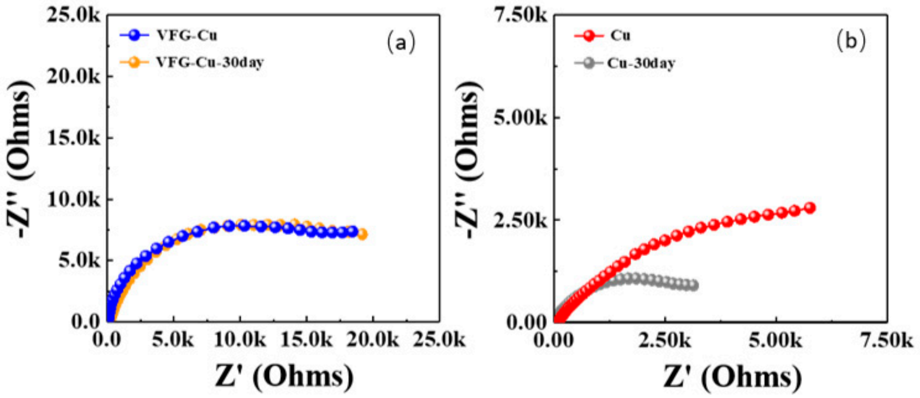

Figure S1. Nyquist plots for (a) VFG-Cu and (b) Cu after exposure to 3.5% NaCl solution for 1 month.
